# Prostatic abscess in a patient with ST-elevation myocardial infarction: a case report

**DOI:** 10.1186/s12872-016-0228-0

**Published:** 2016-02-19

**Authors:** Yoshito Kadoya, Tsuneaki Kenzaka

**Affiliations:** Department of Internal Medicine, Kyotambacho Hospital, Kyotambacho, Kyoto, Japan; Division of Community Medicine and Career Development, Kobe University Graduate School of Medicine, Kobe, Japan

**Keywords:** Prostatic abscess, ST-elevation myocardial infarction, Catheter-associated urinary tract infection, Urinary tract infection, Indwelling bladder catheter

## Abstract

**Background:**

In patients with ST-elevation myocardial infarction (STEMI), urinary tract infection is the most common infection-related complication. Prostatic abscess in a patient with STEMI is very rare.

**Case presentation:**

We report the case of a 49-year-old Japanese man who developed fever and shaking chills during hospitalization for STEMI. We initially diagnosed catheter-associated urinary tract infection. However, subsequent contrast-enhanced computed tomography revealed multiple large abscesses in his prostate. We decided to treat with antimicrobial agents alone because the patient was receiving dual-antiplatelet therapy and discontinuation is very high risk for in-stent thrombosis. The patient recovered remarkably after treatment without drainage or surgery.

**Conclusions:**

Here, we described the world’s first reported case of prostatic abscess in an immunocompetent patient with STEMI. Early removal of indwelling bladder catheters in patients with STEMI receiving dual-antiplatelet therapy is important to avoid development of prostatic abscess. Furthermore, unnecessary invasive instrumentation should be avoided or limited to diminish the risk of infections.

## Background

The infection-related complication rate in patients hospitalized for ST-elevation myocardial infarction (STEMI) has been reported to be 16.6 % [1]. Urinary tract infection (UTI) is the most common infection-related complication, reported in approximately 6 % of patients with STEMI [1]. Risk factors associated with UTI in such patients include urinary tract catheterization and several comorbidities, such as diabetes mellitus and chronic kidney disease [1]. Here, we report a rare case of prostatic abscess in an immunocompetent patient hospitalized for STEMI.

## Case presentation

**Fig. 1:**
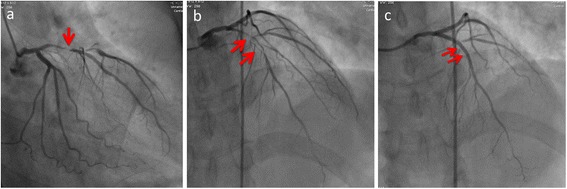
Coronary angiogram of left coronary artery. **a** Right anterior oblique (RAO)-Caudal view, pre-percutaneous coronary intervention (PCI), (**b**) Anteroposterior (AP)-Cranial view, pre-PCI, (**c**) AP-Cranial view, post-PCI. Red arrows are culprit lesion

A 49-year-old Japanese man complaining of chest pain was taken by ambulance to our hospital. His medical history included surgery for inguinal hernia at 45 years of age. He also had a history of smoking with a Brinkman index score of 300. Electrocardiography showed ST elevation in leads V2-5. Cardiac biomarkers were also elevated as following: high sensitive Troponin I, 0.067 ng/ml (standard value: 0.00–0.03 ng/ml). Based on these findings, we diagnosed STEMI, and performed emergent coronary angiography, which showed occlusion of the left anterior descending artery (Fig. 1a, b). Thus, we performed emergent percutaneous coronary intervention (PCI) (Fig. 1c). After surgery, the patient was moved to the intensive care unit with an indwelling bladder catheter, where he recovered without any acute complications.

**Fig. 2:**
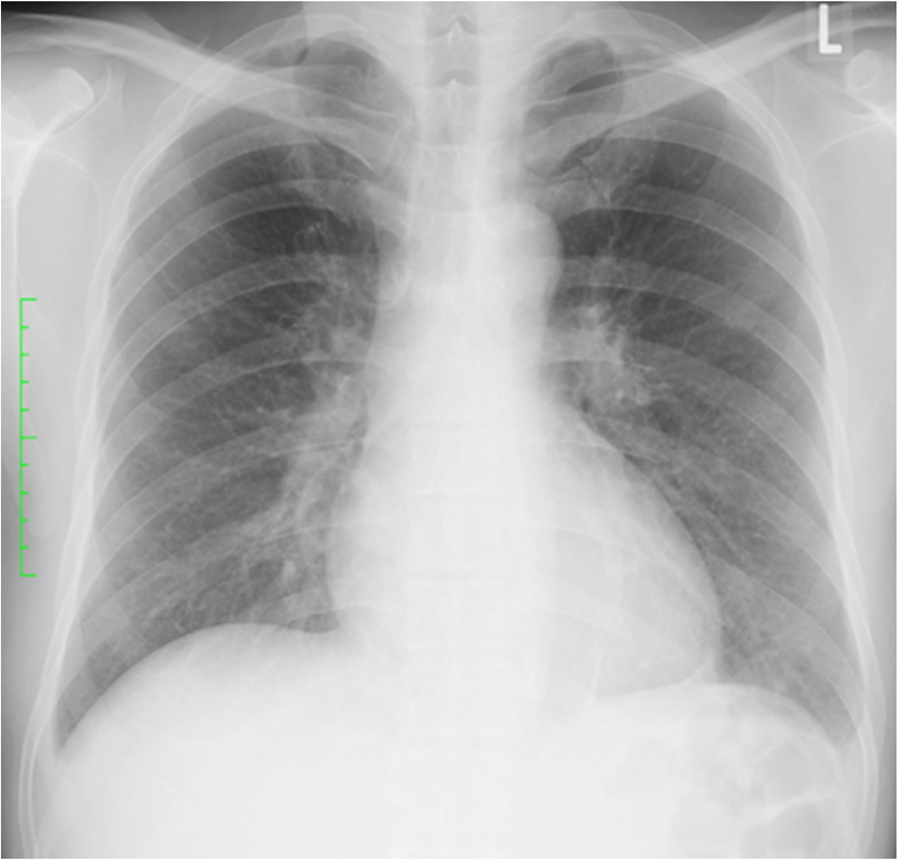
Chest radiograph, showing no infiltrative shadows

**Fig. 3:**
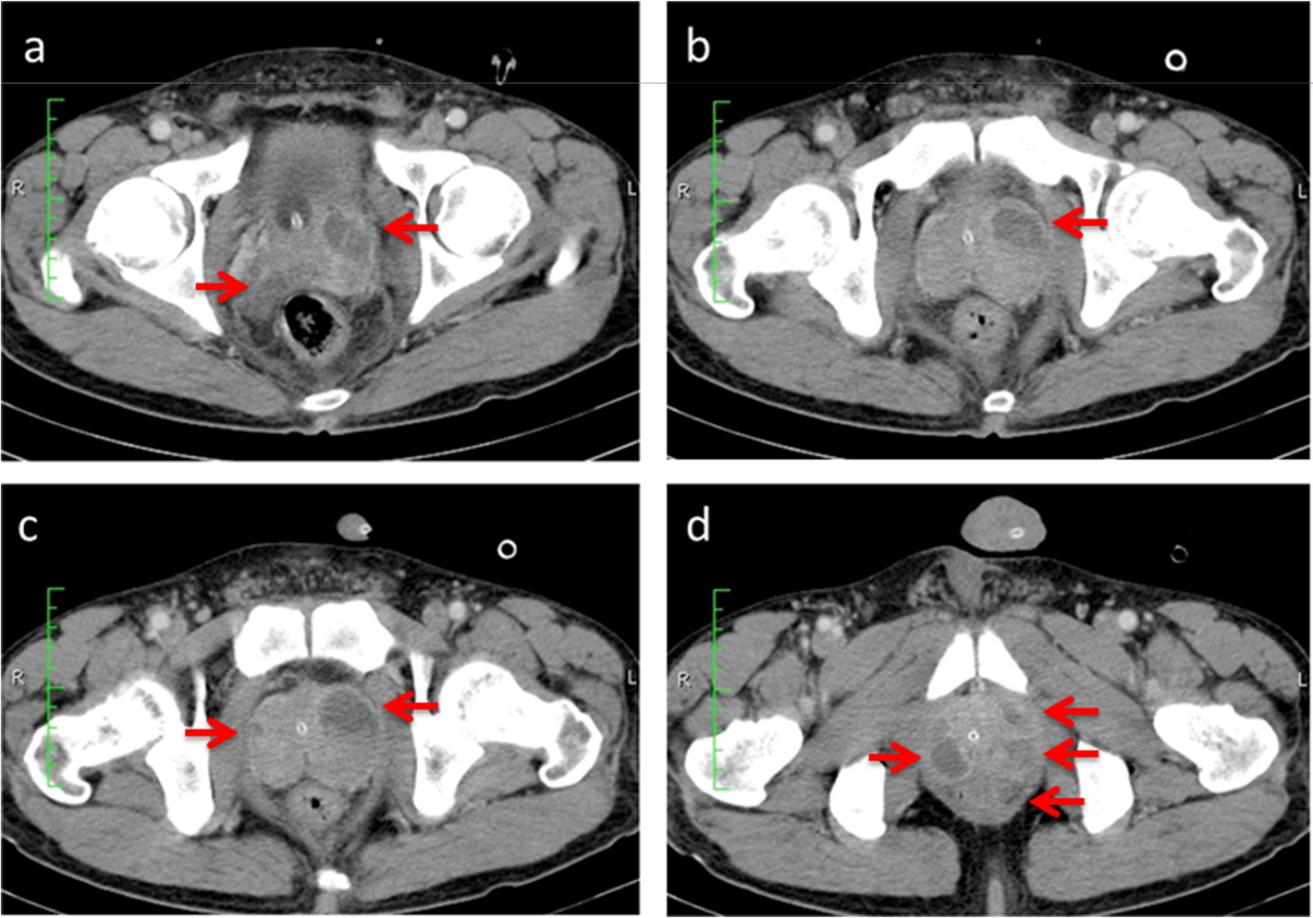
Contrast-enhanced computed tomography scan, revealing multiple large prostatic abscesses (red arrows). In sequence from (**a**) to (**d**), the images are sliced from the head side to the foot side

Three days after PCI, the patient developed fever and shaking chills. He had a body temperature of 39.2 °C, blood pressure of 103/68 mmHg, regular pulse rate of 103 beats/min, respiratory rate of 18 breaths/min, and oxygen saturation of 97 % (without oxygen administration). His heart and breath sounds were normal, and his abdomen was soft and flat with no tenderness. There was no skin rash, and joint findings were normal. Laboratory data at onset of fever and shaking chills are shown in Table 1. Notably, he had a white blood cell count of 13,760 cells/mm^3^, hemoglobin level of 12.4 g/dL, and C-reactive protein level of 8.29 mg/dL. Cardiac enzymes were still elevated due to the STEMI, but were returning toward normal. Chest radiography showed no infiltrative shadows (Fig. 2). Urinary Gram staining revealed middle-sized gram-negative rods that were phagocytized by leukocytes. We initially diagnosed catheter-associated UTI. Blood and urine cultures were performed, his indwelling bladder catheter was removed, and he was initially administered intravenous (IV) cefmetazole (1 g every 8 h). The fever was brought down temporarily, but fever developed again at 4 days after starting the antimicrobial agent. In addition, micturition and pain while urinating persisted. Blood and urine cultures were both positive for *Pseudomonas aeruginosa*. We suspected abscess formation, and performed contrast-enhanced computed tomography, which showed multiple large abscesses in his prostate (Fig. 3).

**Table 1:** Laboratory data at onset of fever and shaking chills

Parameter	Recorded value	Standard value
White blood cell count	13.76 × 10^9 /L	4.00–7.50 × 10^9 /L
Hemoglobin	12.4 g/dL	11.3–15.2 g/dL
Hematocrit	37 %	36–45 %
Platelet	184 × 10^9 /L	130–350 × 10^9 /L
C-reactive protein	8.29 mg/dL	≦0.14 mg/dL
Total protein	6.2 g/dL	6.9–8.4 g/dL
Albumin	3.4 g/dL	3.9–5.1 g/dL
Total bilirubin	0.8 mg/dL	0.4–1.5 mg/dL
Aspartate aminotransferase	72 U/L	11–30 U/L
Alanine aminotransferase	41 U/L	4–30 U/L
Lactate dehydrogenase	402 U/L	109–216 U/L
Alkaline phosphatase	192 U/L	107–330 U/L
γ-glutamyltranspeptidase	19 U/L	<70 IU/L
Creatinine	0.79 mg/dL	0.63–1.03 mg/dL
Sodium	137 mEq/L	136–148 mEq/L
Potassium	4.1 mEq/L	3.6–5.0 mEq/L
Glucose	113 mg/dl	70–109 mg/dl
B-type natriuretic peptide	107 pg/mL	<20 pg/mL
Urinary protein	+	-
Urinary occult blood	+++	-
Urinary nitrite	+	-
Urinary white blood cell	20–29/high-power field	-
Urinary red blood cell	50–99/high-power field	-

After consulting a urology specialist about drainage or surgical treatment, we decided to treat with antimicrobial agents alone because the patient was receiving dual-antiplatelet therapy and discontinuation is very high risk for in-stent thrombosis. We administered IV meropenem (1 g every 12 h) and amikacin (200 mg every 12 h) for 2 days, and then changed to levofloxacin (750 mg per day) based on drug sensitivity. Blood cultures were negative on the 10th day after onset of fever and shaking chills, while contrast-enhanced computed tomography showed the abscess was decreasing on the 17th day. The patient was discharged on the 27th day of hospitalization. He had in total 6 weeks of antimicrobial therapy, and showed remarkable recovery. We continue to see this patient regularly at our clinic, and he has been in good health for more than 3 years.

## Discussion

We experienced a case of prostatic abscess in a patient hospitalized for STEMI. In such patients, UTI is a frequent complication, whereas prostatic abscess is very rare. To the best of our knowledge, this is the world’s first reported case of prostatic abscess in an immunocompetent patient with STEMI.

Prostatic abscess is thought to occur as a consequence of inadequately treated acute bacterial prostatitis [2]. In recent years, prostatic abscess has rarely been reported due to the widespread use of antibacterial agents. Furthermore, it has been noted that only 2.7 % of cases of acute bacterial prostatitis will develop into prostatic abscess [3]. Based on the fact that acute bacterial prostatitis is diagnosed in approximately 0.5 to 2.5 % of patients with prostate symptoms [4], we can assume that prostatic abscess accounts for a very small percentage of all UTIs.

The most common pathogen associated with prostatic abscess is *Escherichia coli*. However, unusual pathogens, such as *Staphylococcus aureus*, *Pseudomonas aeruginosa*, *Mycobacterium tuberculosis*, and *Candida,* also have been reported [5, 6]. Prostatic abscess usually occurs in immunosuppressed patients with such conditions as diabetes mellitus, chronic renal failure, hemodialysis, cancer, cirrhosis, or human immunodeficiency virus infection [7–10]. Other predisposing factors include indwelling bladder catheter, instrumentation of the lower urinary tract, bladder outlet obstruction, and biopsy of the prostate [7–10]. In the present case, the patient was not immunosuppressed. Therefore, the indwelling bladder catheter at the time of emergent PCI for STEMI was recognized as the main cause of his prostatic abscesses.

Currently, drug-eluting stents are commonly used in PCI for acute myocardial infarction. Such patients should receive dual-antiplatelet therapy after drug-eluting stent implantation. In the present case, prostatic abscess drainage, which should be done in many cases, was not performed because the patient was receiving dual-antiplatelet therapy. Because patients who undergo PCI usually receive dual-antiplatelet therapy, they have a higher risk of hemorrhagic complications with drainage or surgical treatment for an abscess. Thus, it is important to avoid unnecessary indwelling bladder catheters, or to promptly remove those that are no longer required, so that patients can avoid development of prostatic abscess. Furthermore, for all the patients, unnecessary invasive instrumentation should be avoided or limited as much as possible to diminish the risk of infections.

## Conclusions

In conclusion, we described the world’s first reported case of prostatic abscess in an immunocompetent patient STEMI. Such patients usually have a higher risk with drainage due to receiving dual-antiplatelet therapy. Early removal of indwelling bladder catheters is important to avoid development of prostatic abscess. Unnecessary invasive instrumentation should be avoided or limited to diminish the risk of infections.

### Consent

Written informed consent was obtained from the patient for publication of this Case report and any accompanying images. A copy of the written consent is available for review by the Editor of this journal.

## Abbreviations

IV: intravenous
PCI: percutaneous coronary intervention
STEMI: ST-elevation myocardial infarction
UTI: urinary tract infection
